# N-acetylcysteine improves the inhibitory effect of Quercetin-rich onion extract on HT-29 and HCT-116 colorectal cancer migration and invasion through iNOS suppression

**DOI:** 10.7150/ijms.86573

**Published:** 2023-07-24

**Authors:** Rataya Tanomrat, Chonnapat Naktubtim, Parichaya Aimvijarn, Prasit Suwannalert

**Affiliations:** 1 Department of Pathobiology, Faculty of Science, Mahidol University, Bangkok 10400, Thailand.; 2 Pathobiology Information and Learning Center, Department of Pathobiology, Faculty of Science, Mahidol University, Bangkok 10400, Thailand.; 3 Department of Pathology, Faculty of Medicine, Kasetsart University, Bangkok 10900, Thailand.

**Keywords:** quercetin, N-acetylcysteine (NAC), iNOS, migration, invasion, colorectal cancer.

## Abstract

As colorectal cancer (CRC) usually presents at an advanced stage, it responds poorly to traditional surgery and chemoradiotherapy. Reactive oxygen species (ROSs) are a critical factor in cancer progression. Quercetin, a bioflavonoid derived from onion peel extract, provides great anti-oxidant and anti-cancer potential. Therefore, quercetin in combination with N-Acetylcysteine (NAC), a well-known anti-oxidant and adjuvant agent in cancer-chemotherapeutic drugs, was considered as a way of increasing treatment efficacy. Thus, this study aimed to evaluate the improvement effect of quercetin in combination with NAC in human CRC (HT-29 and HCT-116) cell progression, migration and invasion. Firstly, the effects of quercetin, NAC, and the combination of quercetin and NAC on cellular oxidants and glutathione levels were evaluated. Cell viability, anti-migrative activity and invasive activity were determined by MTT, wound healing, and Matrigel invasion tests, respectively. Then, the proteins involved in cell migration, invasion, and cellular oxidants were investigated. Moreover, the gene expression and overall survival were further validated by the GEPIA2 database. The results reveal that the combination was most effective in decreasing cellular oxidants and increasing glutathione levels, while there was a significant decrease in cancer cell migration and invasion involved in the suppression of iNOS, ICAM-1, and MMP-2 proteins. Furthermore, bioinformatic analysis verified that iNOS, ICAM-1, and MMP-2 were highly expressed in CRC tissue and also associated with a poor prognosis. This study demonstrated that Quercetin has higher efficacy when used in combination with NAC, representing a potential combination agent for anti-cancer drug development.

## Introduction

Colorectal cancer **(**CRC) is consistently recorded as one of the top three causes of mortality worldwide 1,2. Evidence shows that in the last stage of CRC, metastasis significantly reduces the survival opportunities for the patients 1. Metastasis is when cancer cells disseminate to other organs. Cancer cell migration and invasion are essential processes of cancer metastasis 3,4. Interestingly, nitric oxide (NO) is a free radical that plays a vital role as a messenger molecule to stimulate and induce cancer cell progression, especially cancer migration, invasion, angiogenesis, and metastasis 5. An increase in NO is directly related to the overexpression of the NO synthesis enzyme, nitric oxide synthase (NOS). Inducible NOS (iNOS) and endothelial NOS (eNOS) have been recognized as the cancers targeted to promote the accumulation of NO-related stress-induced migration, invasion, and metastasis 5-7. The role of glutathione (GSH) is to act as an antioxidant/radical scavenger to redox the signaling modulator. When there is an overproduction of NO, it can be scavenged by the activity of GSH to form GSNO molecules and be excreted out of the cell 8. During the processes, cancer cells have been observed to overexpress the various molecules, especially the mobility molecules and ECM degradation enzymes that allow cancer cells to move and penetrate the surrounding tissue 9-11. Intercellular adhesion molecule-1 (ICAM-1) is a transmembrane glycoprotein recognized as an adherent molecule that supports cell movement in a variety of cell types such as leukocytes and endothelial cells. In addition, ICAM-1 plays an important role in supporting cancer malignancy. One previous study reported that the high migration ability of cancer is associated with the overexpression of ICAM-1. Therefore, the ICAM-1 acts as an anti-cancer-targeted molecule 9-12. Matrix metalloproteinases (MMPs) are the major enzymes in tissue repair that degrade the extracellular matrix. The upregulation of MMPs, including MMP-2, 7, and 9, is found in most cancers during the invasive process 10. Among the MMP family, MMP-2 was specified as a major enzyme of colorectal cancer that degraded the basal membrane and ECM, allowing cancer cells to penetrate the nearby tissue 13. The use of natural extracts has been focusing on their cancer therapeutics effects. Quercetin, a natural flavonoid, is present in various vegetables, fruits and their derivative products, such as kale, red wine, green tea, berries, and especially onions 15. The quercetin-rich onion peel extract (QOE) also provides great potential for inhibiting cancer progression, including migration and invasion 14,15. However, the effect of the extract is limited due to its properties, such as poor bioavailability, burst action, poor absorption, and rapid systemic degradation 16. Therefore, improving the effect of the extract has been proposed, with N-acetylcysteine (NAC) put forward as a potential improvement agent. The anti-oxidant function of NAC is already well-known as an adjuvant agent in cancer-chemotherapeutic drugs 17,18. A clinical examination also represents an opportunity to improve cancer treatment through the combination of NAC and currently used drugs 17. Therefore, this study aimed to investigate the improvement effect of NAC on QOE in suppressing colorectal cancer migration and invasion, through the ICAM-1 and MMP-2 associated with iNOS/eNOS suppression. In addition, the iNOS, ICAM-1, and MMP-2 gene expressions and overall survival were further validated by the GEPIA2 database.

## Methods and Materials

### Reagents and antibodies

Quercetin was extracted from onion peel (Detox (Thailand) Co., Ltd., Chiangmai, Thailand), dissolved in dimethyl sulfoxide (DMSO; Sigma-Aldrich, MO, USA), and stored at -20 °C. Quercetin standard and N-Acetylcysteine (NAC) were obtained from Sigma-Aldrich (USA). Inducible nitric oxide synthase (iNOS, 1:500 dilution), endothelial nitric oxide synthase (eNOS, 1:500 dilution), matrix metallopeptidase 2 (MMP-2, 1: 5000 dilution), and intercellular adhesion molecule 1 (ICAM-1, 1: 5000 dilution) were all obtained from Abcam (UK).

### Cell culture and treatment

Human colorectal cell lines, HT-29 cells (ATCC^®^ HTB-38™, Virginia, USA), and HCT-116 cells (ATCC^®^ CCL-247™, Virginia, USA) were cultured in McCoy's 5A (Modified) Medium and then supplemented with 10% heat-inactivated fetal bovine serum (FBS), 1% of L-glutamine, 1% of penicillin-streptomycin and non-essential amino acid. The cells were incubated at 37 °C in a humidified atmosphere supplied by a 5% CO_2_ incubator (Lab-line; USA).

### 3-(4,5- dimethylthiazol-2-yl)-2,5-diphenyl-tetrazolium bromide (MTT) assay

The cell viability was evaluated as mentioned in previous studies 19,20. The HT-29 and HCT-116 cells were seeded and maintained in 96-well plates for 24 h. Next, the culture media were removed and replaced by media containing samples of various concentrations. The treated cells were incubated for 24 h, and the culture media were removed, after which, new media containing MTT solution were added and the cells incubated for 2 h. After incubation, the cells were replaced by dimethyl sulfoxide (DMSO). The results were determined using an automated microplate reader (1420 Victor 2, Wallac, USA) at 570 nm.

### DCFH-DA

Intracellular oxidants were detected with DCFH-DA in a modified method 21. DCFH-DA is cleaved in the intracellular cell by nonspecific intracellular esterase and it then turns to highly fluorescent 2,7-dichlorofluorescein (DCF) upon oxidation by free radicals. Briefly, the HT-29 and HCT-116 cells were seeded in 96-well plates and incubated at 37 °C in a humidified atmosphere in a 5% CO_2_ incubator for 24 h. The cells were then treated with the sample for 1 h. Next, the cultured media were removed and replaced with 10 µM of DCFH-DA media, and then incubated at 37 °C in a humidified atmosphere in a 5% CO_2_ incubator for 1 h. In this process, DCF fluorescence intensity was assessed for cellular oxidant as stress inside the cell at excitation/emission wavelengths of 485/535 nm by using a fluorescence microplate reader (The Spark® multimode microplate reader, TECAN, Switzerland). N-acetyl cysteine (NAC) was used as a positive control.

% Cellular oxidant = [(O.D. sample - O.D. blank) / (O.D. control - O.D. blank)] x 100

The result was determined as the percentage of cellular oxidant (% cellular oxidant) as stress inside the cell.

### GSH+GSSG/GSH assay kit (colorimetric) for reduced form, GSH and total glutathione level

The level of cellular glutathione including the reduced (GSH) form and total glutathione were measured by GSH+GSSG/GSH assay kit (colorimetric) from Abcam (UK). Briefly, the HT-29 and HCT-116 cells were seeded in 96-well plates and incubated at 37 ˚C in a humidified atmosphere in a 5% CO_2_ incubator for 24 h. After incubation, the culture media were removed and replaced with the media containing the sample and then incubated at 37 ˚C in a humidified atmosphere in a 5% CO_2_ incubator for 24 h. Next, cells were added to the reaction mix and incubated at room temperature for 10 min to generate NADPH. For detecting GSH only, glutathione reductase was omitted from the reaction mix. Afterwards, cells were added to the GSH standard or the sample solution and the plate was incubated at room temperature for 5-10 min; then the substrate solution was added to the pate and incubated at room temperature for 5-10 min. The result was determined by using an automate microplate reader (1420 Victor 2, Wallac, USA) at 405 nm.

Total glutathione = [(O.D. samples - O.D. blank) / Slope STD Curve

### Wound healing assay

Cell invasion assay was used to analyze the ability of malignant cells to respond directionally to various chemoattractant invading normal surrounding tissues by using a modified method 22. The HT-29 and HCT-116 cells were seeded in 6-well plates and incubated until 90% confluent. The cells were then wounded by scratching lines in them with an SPLScarTM Scratcher (SPL, Korea) and washed with PBS buffer. After that, the cells were treated with samples of various concentrations and incubated for 24 h. Finally, the results were obtained after 0 and 24 h by measuring the area of the wound region lacking cells under the microscope. Photographs were taken using the ImageJ program.

### Matrigel invasion assay

Cell migration was performed by using Matrigel invasion assay with a modified method 22. Cell culture inserts (Corning, USA) with 8.0 µm pores were coated with extracellular matrix (ECM) gel from Engelbreth-Holm-Swarm murine sarcoma (Sigma-Aldrich, USA). The HT-29 and HCT-116 cells were seeded on the upper part of the inserts and media containing 30% fetal bovine serum (FBS) were added in the lower part of the 24-well plates, which were then incubated for 24 h. The cultured media were removed and replaced with media containing the samples of various concentrations, which were then incubated for 72 h for the HT-29 cells and 120 h for the HCT-116 cells. The invaded cells were determined by removing the non-invading cells from the upper surface of the insert part and washing them with PBS buffer. Next, the lower part of the insert was stained with hematoxylin and eosin (H&E) before the number of invading cells was investigated under the microscope. The results were expressed as the number of invasion cells in 10 high power fields (HPF).

### Western blotting

The quantification of protein expressions was determined by using the Western blot analysis method modified from previous works 23. The HT-29 and HCT-116 cells were seeded into 6-well plates and maintained for 24 h. After incubation, the cultured media were removed and replaced with media containing the samples of various concentrations. Then, the cultured media were removed and washed with an ice-cold PBS buffer, pH 7.4. Next, the cells were lysed with a lysis buffer containing a RIPA lysis buffer (Merck, Germany) and complete protease inhibitor (Sigma-Aldrich, USA) and then homogenized using a homogenizer (Vibro cell, Sonics, USA). After that, the cell lysates were centrifuged at 8,000 rpm. at 4 ºC for 10 min to obtain the supernatant. The total protein was then determined using protein kits (Merck, Germany) in accordance with the commercial instructions. The protein in each sample was stained with a Laemmli loading buffer (Merck, Germany) and electrophoresed in 12% SDS-PAGE and transferred overnight to polyvinylidene difluoride (PVDF) membranes (Millipore, Germany). The membranes were washed with TBST and blocked with 5% skimmed milk at room temperature. After blocking, the membranes were treated with a primary antibody at 4 ºC for 24 h and washed with TBST. The membranes were then treated with a secondary antibody at room temperature for 1 h, washed with TBST and incubated with Luminata Forte western HRP substrate (Merck, Germany) for 5 min. Finally, the proteins were determined using a gel documentation analyzer (ChemiDoc MP Imaging System, Bio-Rad, USA) and the densitometry was analyzed by using the Image J software.

### Gene expression profiling interactive analysis (GEPIA)

The expression of MMP2 and ICAM1 was performed by using online software, Gene Expression Profiling Interactive Analysis (GEPIA), based on 275 tumors and 349 normal samples from the TCGA (The Cancer Genome Atlas) and the Genotype-Tissue Expression project (GTEx). GEPIA was used to compare the expression of MMP2 and ICAM1 in the tumor and normal tissues in patients with colon adenocarcinoma (COAD). The overall survival patient analysis was performed based on gene expression.

### Statistical analysis

Statistical differences from each experimental test condition's respective controls were analyzed as mean ± standard deviation (mean±SD) and compared by ANOVA (one-way analysis of variance) or the t-test. p≤0.05 represented a significant difference. GraphPad Prism version 9 (GraphPad Software Inc., CA, USA) was used to perform all statistical analyses.

## Results

### Effects of quercetin extract, NAC, and quercetin in combination with NAC on colorectal cancer cell viability

**Figure 1:**
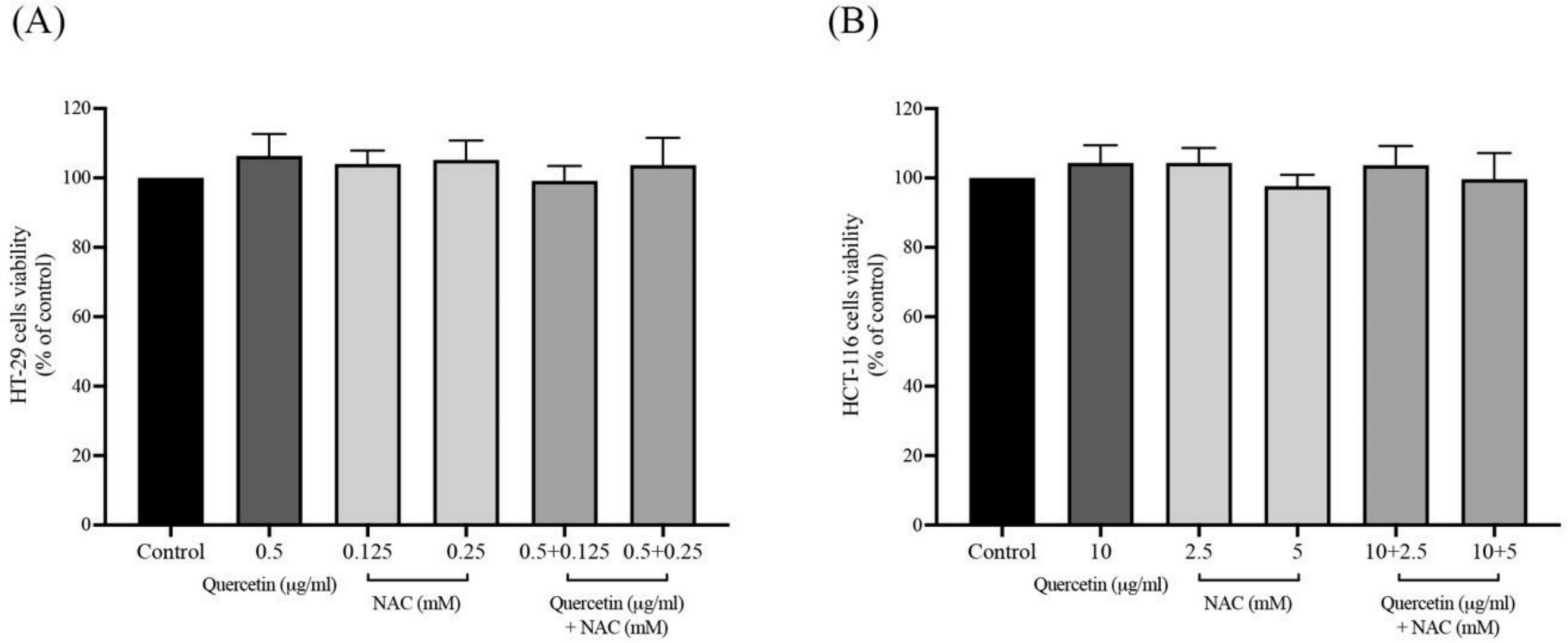
The cell viability of quercetin, NAC, and combination treatment on HT-29 **(A)** and HCT-116 **(B)**. The results were expressed as mean±SD.

The cell viability of quercetin, NAC, and quercetin combined with NAC was analyzed using the 3-(4,5-dimethylthaizol-2-yl)-2,5-diphenyltetrazolium bromide (MTT) assay to provide non-cytotoxic concentrations of the samples. The HT-29 cells were treated with 0.5 μg/ml of quercetin, 0.125 mM and 0.25 mM of NAC, and 0.5 μg/ml of quercetin combined with 0.125- and 0.25-mM NAC (Figure 1A). The HCT-116 cells were treated with 10 μg/ml of quercetin, 2.5 mM and 5 mM of NAC, and 10 μg/ml of quercetin combined with 2.5- and 5-mM NAC (Figure 1B). These concentrations of quercetin, NAC, and quercetin combined with NAC were considered safe doses for analyzing their effects on cells.

### Effects of quercetin, NAC, and its combination treatment on cellular oxidants in cancer cells

**Figure 2:**
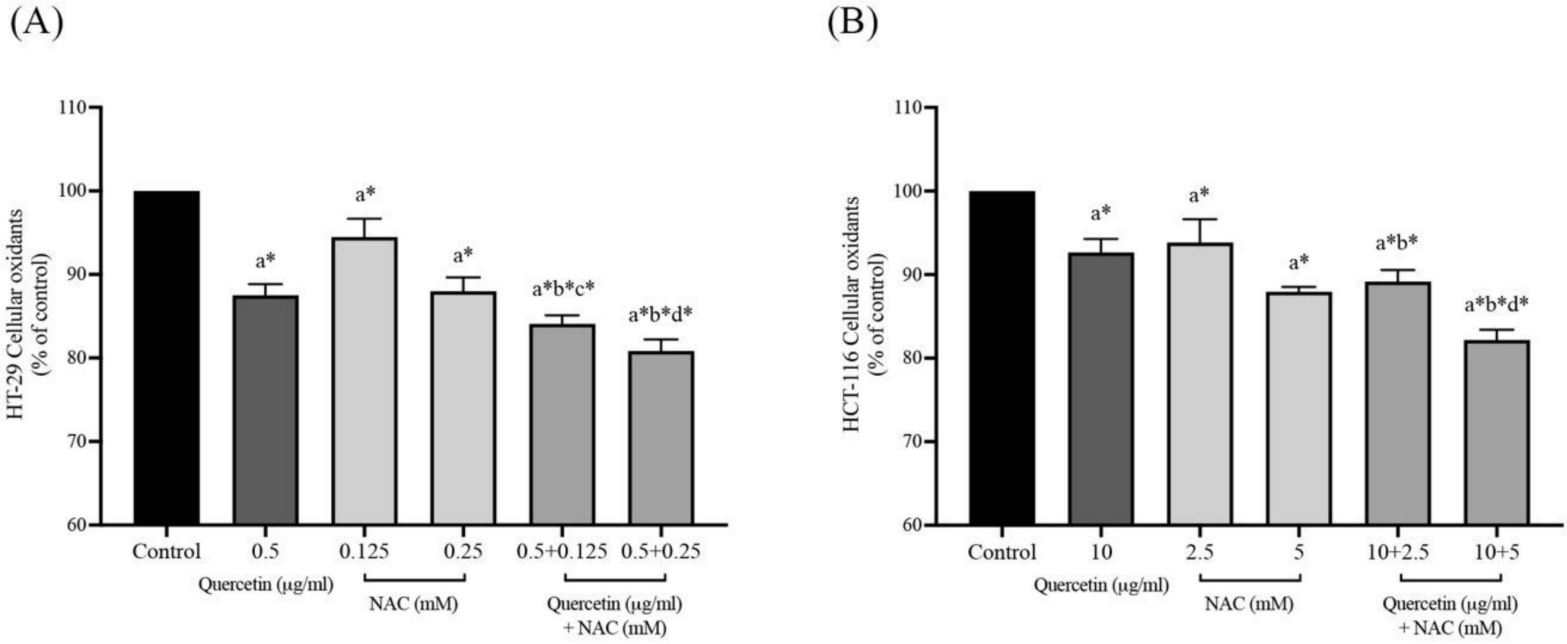
Cellular oxidants in HT-29 **(A)** and HCT-116 **(B)** colorectal cancer cells after being treated with quercetin and a combined treatment. Results were expressed in a bar graph showing cellular oxidant levels. The results were expressed as mean±SD. *Statistical significance was at p≤0.05; a*, b*, c*, d* compared with the control, 0.5 or 10 μg/ml quercetin, 0.125- or 2.5-mM NAC and 0.25- and 5-mM NAC in HT-29 and HCT-116, respectively.

After the safe doses of quercetin, NAC, and combined treatment were confirmed by MTT assay, at the same concentrations, the intracellular oxidants were continuously detected by DCFH-DA analysis. As shown in Figures 2A and 2B, treatment with quercetin or NAC significantly decreased the cellular oxidants when compared with the control in both the HT-29 and HCT-116 cell lines. Moreover, in the HT-29 cells, quercetin at concentrations 0.5 μg/ml combined with NAC at concentrations of 0.125 (84.24±1.02%) and 0.25 (81.17±1.00%) mM significantly reduced cellular oxidant levels when compared to treatment with quercetin and NAC alone. Similarly, in the HCT-116 cells, quercetin at concentrations of 10 μg/ml combined with NAC at concentrations of 2.5 (89.16±1.39%) and 5 mM (82.18±1.22%) significantly decreased cellular oxidant levels compared to treatment with quercetin alone. Accordingly, the combined treatment showed higher potential for decreasing cellular oxidant levels than the treatment with only quercetin or NAC.

### Effects of quercetin, NAC, and its combination treatment altered the reduced form, GSH and total glutathione in cancer cells

**Figure 3:**
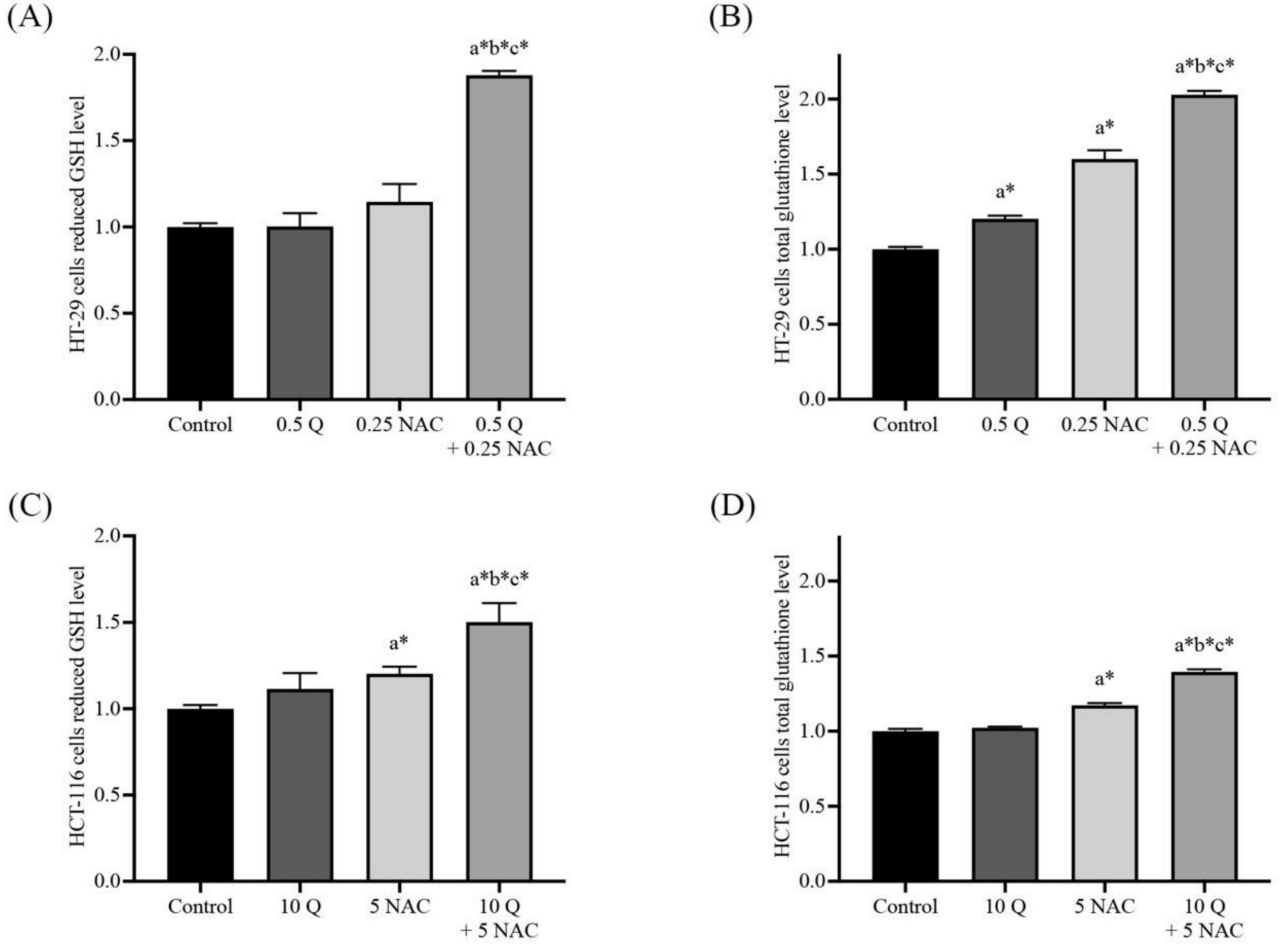
GSH/GSSG ratio in HT-29** (A, B)** and HCT-116 **(C, D)** colorectal cancer cells after being treated with quercetin and a combined treatment. The levels of GSH (A, C) and total glutathione (B, D) were investigated by GSH+GSSG/GSH assay kits. The results were expressed as mean±SD. *Statistical significance was at p≤0.05; a*, b*, c* compared with the control, 0.5 or 10 μg/ml quercetin and 0.25- and 5-mM NAC in HT-29 and HCT-116 cells, respectively.

The levels of cellular glutathione, including reduced form (GSH) and total glutathione, were measured by GSH+GSSG/GSH assay kits in both HT-29 and HCT-116 colorectal cancer cells after being treated with quercetin, NAC, and the combined treatment. The results show that treatment with quercetin and NAC resulted in a slight increase in the reduced form. However, the combined treatments significantly increased GSH levels when compared with the control (Figure 3A and 3C). Likewise, quercetin and NAC treatment also had a lower effect on total glutathione levels than the combination treatment, which increased to 2.03±0.03 and 1.40±0.02 in HT-29 and HCT-116 cells, respectively (Figure 3B and 3D). Thus, the combined therapy displayed a higher potential to increase the level of cellular glutathione than was observed in the cells that were treated with only quercetin or NAC.

### Effects of quercetin, NAC, and its combination treatment on iNOS and eNOS protein expressions

**Figure 4:**
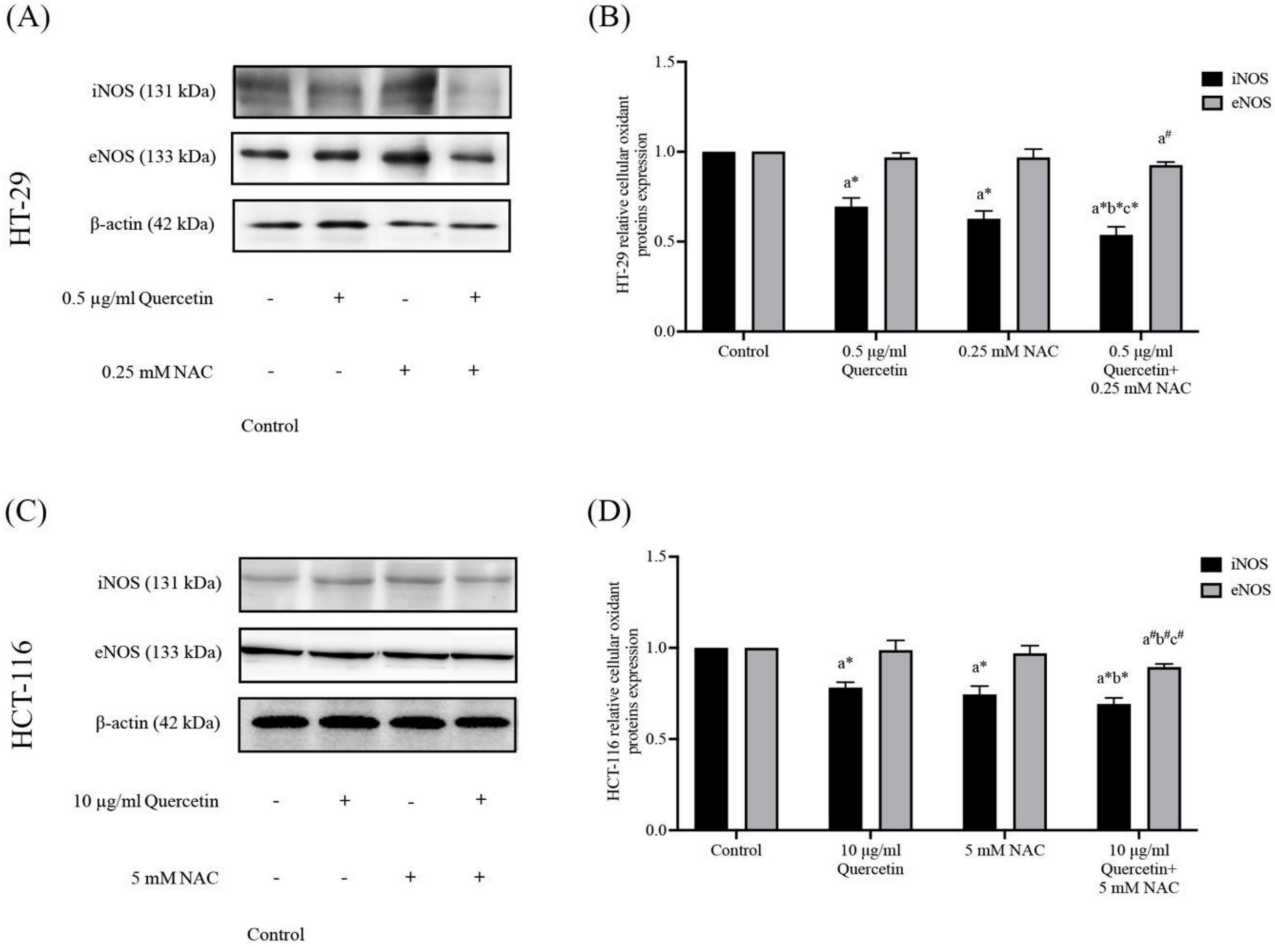
The HT-29 **(A, B)** and HCT-116 **(C, D)** colorectal cancer cells after being treated with quercetin and a combined treatment. The expressions of iNOS and eNOS were investigated by Western blot analysis **(A, C)**. β-actin housekeeping protein was used as the internal control. The band intensities of iNOS and eNOS proteins** (B, D)** were analyzed by the imageJ processing program and the results revealed the relative intensity of β-actin. *Statistical significance was at p≤0.05; For iNOS, a*, b*, c* compared with the control, 0.5 or 10 μg/ml quercetin, and 0.25- and 5-mM NAC in HT-29 and HCT-116, respectively. For eNOS, a^#^, b^#^, c^#^, compared with the control, 0.5 or 10 μg/ml quercetin and 0.25- and 5-mM NAC in HT-29 and HCT-116, respectively.

Western blot analysis was used to determine the nitric oxide synthase (NOS) in HT-29 and HCT-116 cells after being treated with quercetin, NAC, and a combined treatment. In the HT-29, the results show that 0.5 μg/ml quercetin and 0.25 mM NAC suppressed iNOS expression to 0.70±0.05 and 0.63±0.04 respectively when compared with the control (1.00). Interestingly, quercetin combined with NAC showed higher effectiveness in suppressing iNOS to 0.54±0.05 when compared with treatment with quercetin and NAC alone. However, the same concentrations did not affect eNOS expression compared with the control (Figure 4A and 4B). Similarly, for the HCT-116 cells, the results show that concentrations of 10 μg/ml quercetin and 0.25 mM NAC suppressed iNOS expression by 0.78±0.03 and 0.75±0.05, respectively compared with 1.0 of control. Also, quercetin combined with NAC (0.69±0.03) showed higher suppression of iNOS when compared with treatment by quercetin alone, but it did not affect the expression of eNOS compared with the control (Figure 4C and 4D). Therefore, compared to quercetin and NAC alone, the combined treatment has the highest potential for reducing iNOS expression. However, these treatments have no effect on eNOS expression in either cell line.

### Quercetin extract, NAC, and quercetin in combination with NAC suppressed colorectal cancer cell migration

**Figure 5:**
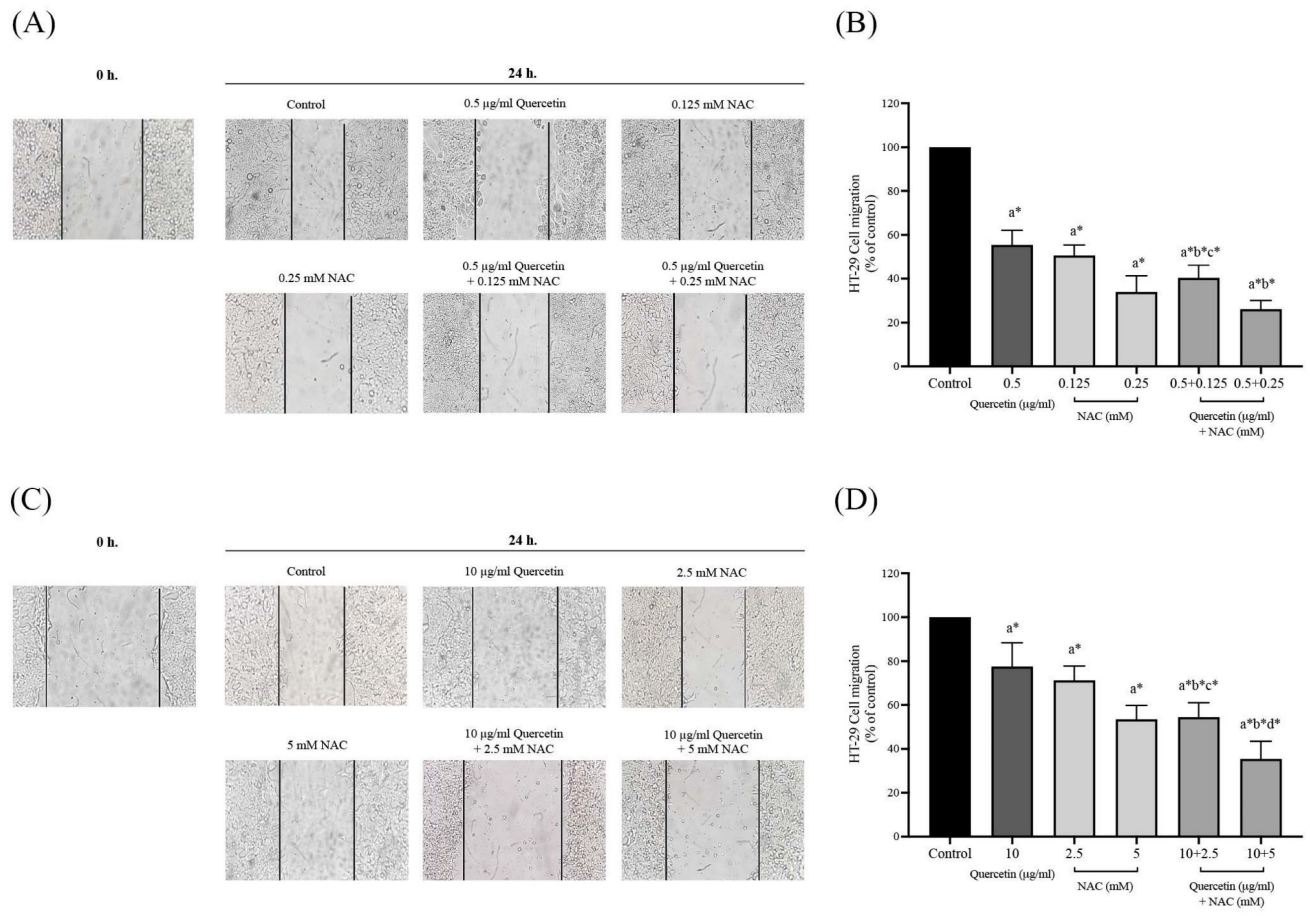
Cell migration in HT-29 and HCT-116 colorectal cancer cells after being treated with quercetin, NAC, and quercetin combined with NAC for 24 h **(A, C)**. Results were expressed in a bar graph as the migrating cells **(B, D)**. The results were expressed as mean±SD. *Statistical significance was at p≤0.05; a*, b*, c*, d* compared with the control, 0.5 or 10 μg/ml quercetin, 0.125- or 2.5-mM NAC, and 0.25- and 5-mM NAC in HT-29 and HCT-116, respectively.

The migration assay of HT-29 and HCT-116 colorectal cancer cells were investigated by wound healing assay. In the HT-29 cells treated with 0.5 μg/ml (55.50±6.67%) of quercetin, the number of migrating cells decreased when compared with the control (100%), while the number of migrating cells decreased more dramatically when treated with 0.5 mg/ml of quercetin combined with 0.125 (40.33±5.85%) and 0.25 mM NAC (26.17±3.97%) (Figure 5A and 5B). The HCT-116 cell results show that treatment with 10 μg/ml quercetin decreased the number of migrating cells to 77.50±10.88% when compared with the control (100%) while the migrating cells decreased more dramatically when treated with 10 μg/ml (51.43±1.72%) of quercetin combined with 2.5 (54.50±6.50%) and 5 mM NAC (35.50±7.97%) (Figure 5C and 5D).

### Quercetin extract, NAC, and quercetin in combination with NAC suppressed colorectal cancer cell invasion

**Figure 6:**
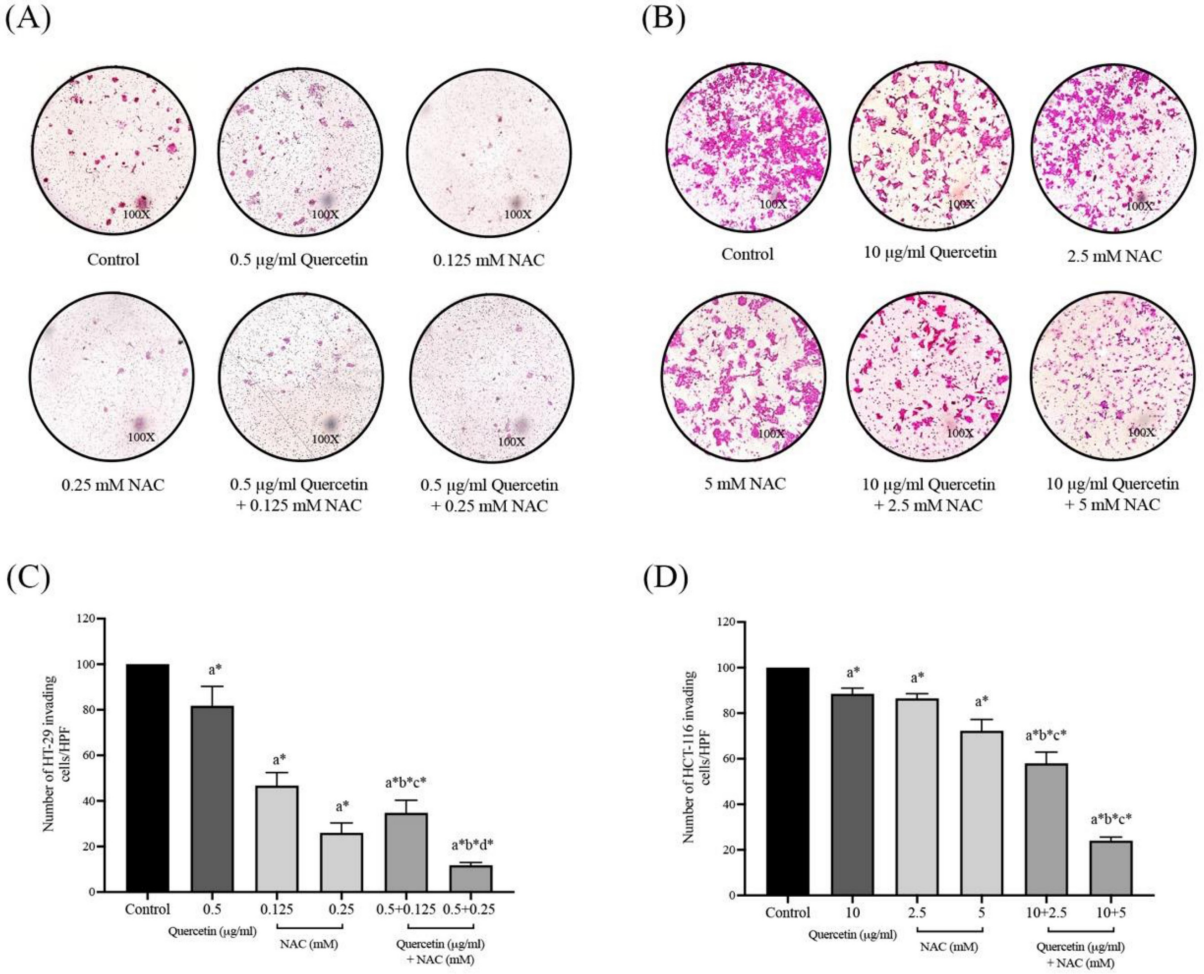
Cell invasion in HT-29 and HCT-116 colorectal cancer cells after treatment with quercetin, NAC, and quercetin combined with NAC for 72 h **(A, B)**. Results were expressed in a bar graph as the invading cells **(C, D)**. The results were expressed as mean±SD. *Statistical significance was at p≤0.05; a*, b*, c*, d* compared with the control, 0.5 or 10 μg/ml quercetin, 0.125- or 2.5-mM NAC and 0.25- and 5-mM NAC in HT-29 and HCT-116, respectively.

The effects of quercetin, NAC, and a combination treatment of quercetin and NAC on HT-29 and HCT-116 colorectal cancer cells were investigated by Matrigel invasion assay. After 72 h, the HT-29 cells treated with 0.5 μg/ml quercetin (81.75±8.54%), 0.125 mM NAC (46.75±5.68%) and 0.25 mM NAC (26.00±4.32%) showed a decreased number of invading cells when compared with the control (100%) and the invading cells decreased dramatically after treatment with 0.5 μg/ml quercetin combined with 0.125 (34.75±5.56%) and 0.25 mM NAC (11.75±1.26%), respectively (Figure 6A and 6C). For the HCT-116 cells, the results show that treatment with 10 μg/ml quercetin (88.50±2.52%), 2.5 mM NAC (86.50±2.08%) and 5 mM NAC (72.25±4.99%) decreased the number of invading cells when compared with the control (100%) and the invading cells decreased dramatically after treatment with 10 μg/ml quercetin combined with 2.5 (58.00±4.90%) and 5 mM NAC (24.00±1.63%) (Figure 6B and 6D). Consequently, the combined treatment reveals the highest efficacy in inhibiting colorectal cancer cell invasion.

### Quercetin extract, NAC, and quercetin in combination with NAC downregulate migrative and invasive cancer cell-related proteins

**Figure 7:**
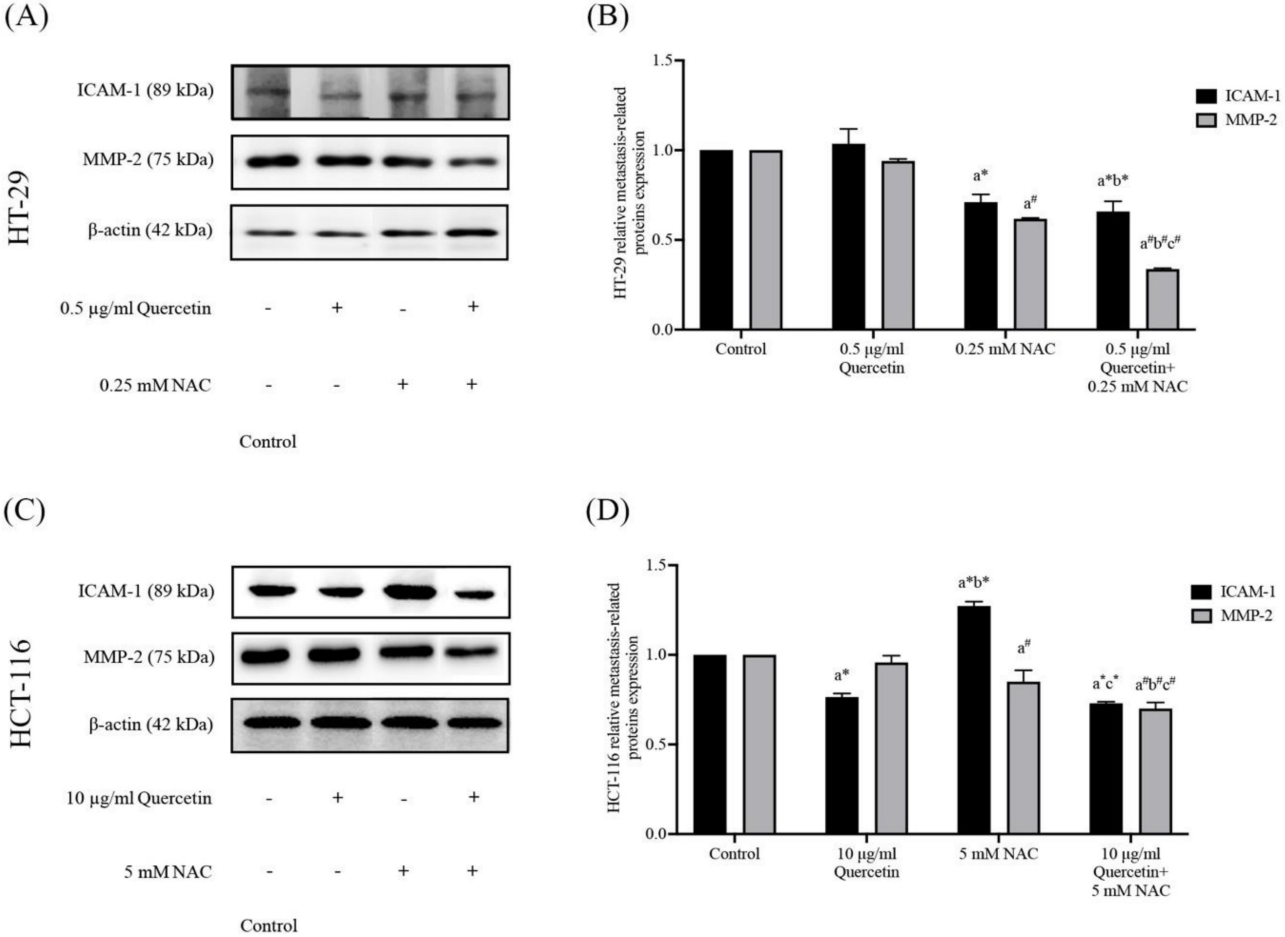
Adhesion molecule (ICAM-1) and degradation enzyme (MMP-2) protein expressions in HT-29 **(A, B)** and HCT-116 **(C, D)** colorectal cancer cells after being treated with quercetin, NAC, and quercetin combined with NAC. The expressions of ICAM-1 and MMP-2 were investigated by Western blot analysis **(A, C)**. β-actin housekeeping protein was used as the internal control. The band intensities of the ICAM-1 and MMP-2 proteins **(B, D)** were analyzed by the imageJ processing program and the results are expressed as relative intensity of β-actin *Statistical significance was at p≤0.05; For iNOS, a*, b*, c* compared with the control, 0.5 or 10 μg/ml quercetin and 0.25- and 5-mM NAC in HT-29 and HCT-116, respectively. For eNOS, a^#^, b^#^, c^#^, compared with the control, 0.5 or 10 μg/ml quercetin and 0.25- and 5-mM NAC in HT-29 and HCT-116, respectively.

To further investigate the effects of the quercetin extract alone, NAC, and quercetin extract in combination with NAC, the expression of ICAM-1 and MMP-2 proteins in colorectal cancer cells was analyzed. Western blot analysis was run after 24 h on the treated cells and the control in both the HT-29 and the HCT-116 cells (Figure 7A and 7C). Compared to the control, in the HT-29 cells treated with quercetin in combination with NAC, the protein expression levels of ICAM-1 and MMP-2 reduced significantly to 0.66±0.06 and 0.34±0.01 respectively, showing higher effectiveness than treatment with quercetin and NAC alone (Figure 7B). Similar to the results for the HT-29 cells, the combination treatment of the HCT-116 cells also has greater efficacy in lowering the protein expression levels of ICAM-1 and MMP-2 to 0.73±0.01 and 0.70±0.04, respectively (Figure 7D). Accordingly, combined treatment has more potential in downregulating migrative and invasive-related proteins than quercetin and NAC alone.

### GEPIA dataset showed higher expression of the iNOS gene in CRC patients and is associated with cancer progression genes; ICAM1 and MMP2

**Figure 8:**
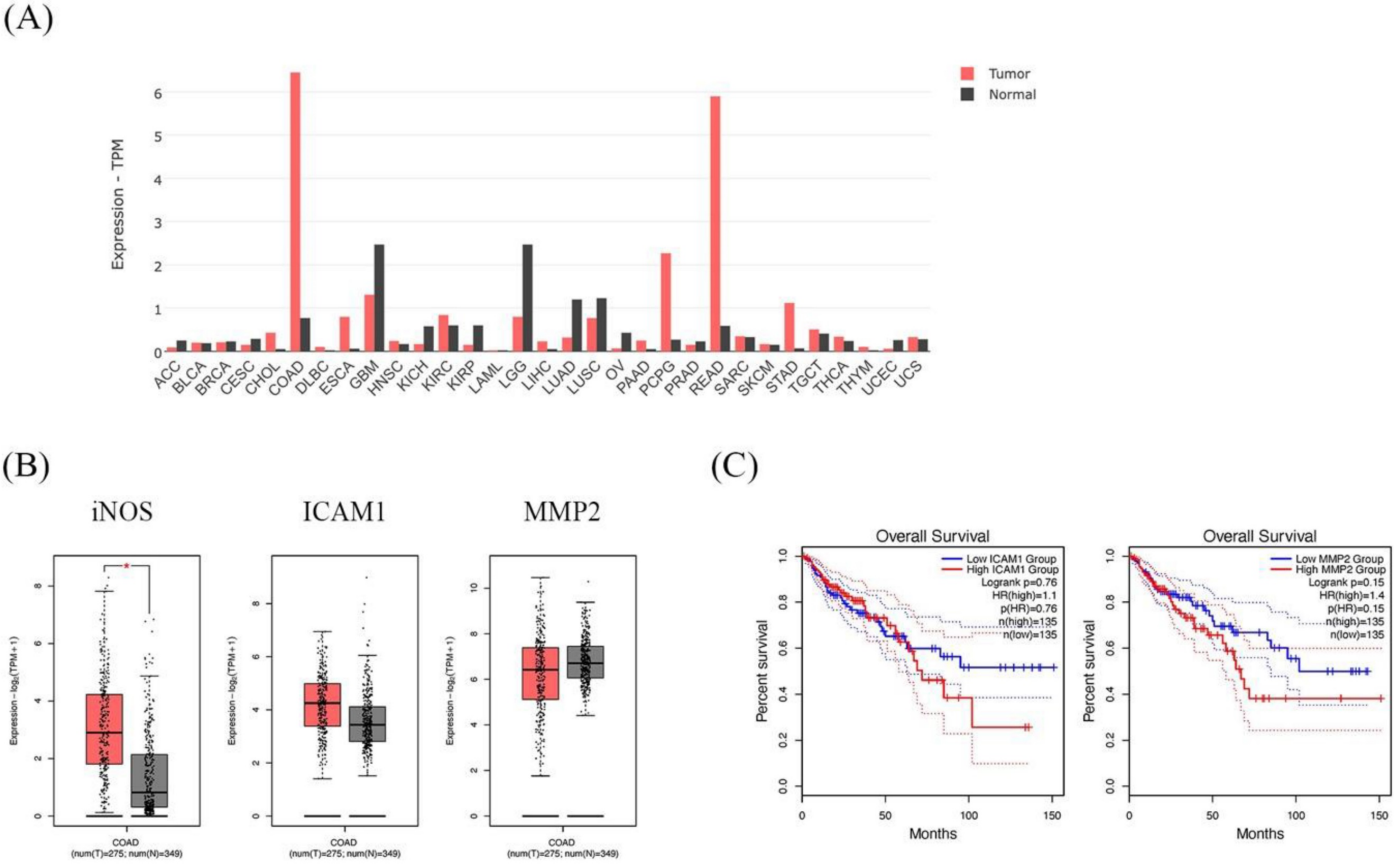
The expression and prognostic effect of iNOS, ICAM1, and MMP2 in Colon Adenocarcinoma (COAD) using the GEPIA database. The bar graph presents the expression profile of iNOS across all tumor samples and paired normal tissues **(A)**. Box plot showing the relative iNOS, ICAM1, and MMP2 expression in COAD patients (red) compared with normal (gray) **(B)**. The survival analysis of ICAM1 and MMP2 in COAD patients **(C)**.

The dataset shows the the iNOS gene expression profile across all tumor samples and paired normal tissues. The COAD in the bar graph represents the highest iNOS gene expression (Figure 8A). In addition, COAD indicated that iNOS was significantly higher in cancer tissues than in normal tissues. Moreover, ICAM1 and MMP2 presented higher expressions of COAD cancer tissues than in normal tissues (Figure 8B). Then, we further analyzed the prognostic effect of ICAM1 and MMP2 in COAD. The expression of ICAM1 and MMP2 showed an association with overall survival in patients. The overall survival of patients with high ICAM1 and MMP2 expressions was lower than those with low ICAM1 and MMP2 expressions (Figure 8C).

## Discussion

Despite the advancement in colorectal cancer (CRC) treatment, many CRC patients still have to overcome undesirable side effects. In particular, advanced CRC with migration, invasion and distant metastases is considered incurable with currently available treatments 24. In this study, plant-derived quercetin extract and NAC were studied for their efficacy in individual and combination treatments. Quercetin is a flavonoid widespread in various types of foods and plants. It is reported to have several beneficial effects on human health such as anti-inflammatory, anti-allergy, anti-viral, and anticancer activities 25. Although a broad range of biological advantages of quercetin were mentioned in this study, its pharmaceutical application and clinical conditions are limited due to its low hydro-solubility, lack of stability in physiologic conditions, and low bioavailability 26. Overcoming the reported problem of quercetin alone may provide short bioavailability. NAC was selected to be combined with quercetin in this work due to previous studies having suggested that NAC treatment effectively reduces the ROS production and ROS-mediated signaling that contribute to cell survival, metastasis, and drug resistance in triple-negative breast cancer (TNBC) cells 27. In addition, NAC has also been suggested as an anti-cancer agent to be used *in vitro* and *in vivo*, either as a stand-alone or as an adjuvant, to decrease the aggressiveness of several cancers 28. Our study shows that both quercetin alone and quercetin combined with NAC offer effective treatment with anti-migration and anti-invasion potential on both CRC cell lines: the HT-29 colon adenocarcinoma cell line, which mutated p53 with high proliferation; and HCT-116, which wild-type p53; with microsatellite instability, which was highly aggressive but with fewer differentiae 29,30. Because HCT-116 cells have higher aggressiveness than HT-29, quercetin and NAC were used in different concentrations for each cell line, with higher doses for HCT-116 cells.

In the first experiment, we studied the effects of quercetin alone and quercetin combined with NAC, with the results confirming the ability to reduce oxidative stress in both cell lines without affecting the cells. This hypothesis was further confirmed in CRC treated by quercetin alone and quercetin combined with NAC, with the results revealing an increase in anti-oxidant levels and a decrease in ROS production. The effects of quercetin on CRC were significant and were more powerful when the CRC was treated with quercetin combined with NAC. These findings indicate that both quercetin and NAC acted as anti-oxidants that could reduce oxidative stress, particularly when NAC was combined with quercetin. Furthermore, our results indicate that in combination with NAC, quercetin exhibits a potent anti-oxidant capacity *in vitro* in both HT-29 and HCT-116 cell lines. There is a large body of information suggesting that overexpression of iNOS and eNOS also enables disease progression 31. The idea that ROS-driven cellular oxidative stress initiates cancer progression has influenced an interest in utilizing anti-oxidants to prevent cancer. Under oxidative stress, iNOS expression is high when there is NO in the cell. Moreover, the study found that iNOS expression shows high expression compared with the treated group in HT-29 and HCT-116. Previous studies also support the view that the overexpression of iNOS can promote NO accumulation, and in order to be executed, the cells need GSH to form GSNO with no toxicity from the cell 8. Surprisingly, quercetin, combined with NAC, shows a high GSH level and can suppress the expression of iNOS. After that, this study focused on the ROS-associated signaling pathways associated with cancer development and progression. There is a direct connection between ROS and epithelial EMT facilitating cancer cells progression. MMP-2, 7 and 9 further corroborate the role of ROS as an important EMT mediator 32. Additionally, ROS also regulates the cell surface protein, ICAM-1, in TNF-α-activated retinal pigment epithelial cells via the NF-κB pathway 33. Another important cellular event involved in cancer initiation and progression is ROS-generating enzymes (e.g., nitric oxide synthase). In addition, our observations indicate that quercetin and quercetin combined with NAC potentially suppressed invasion in both tested CRC cell lines, whereby the invasion protein (MMP-2) in HCT-116 cells does not decrease significantly when treated by quercetin alone but is more sensitive to applied treatments of quercetin combined with NAC. The anti-invasive effects of quercetin in HCT-116 cells are probably based on a cell characteristic: sensitive wt p53 cancer cell lines. Several studies have demonstrated that an alteration of p53 increased the cell motility of fibroblasts, keratinocytes, epithelial cancer lines (HCT-116) and neurons, increasing their growth cone motility associated with the compromised p53 function 34-37 that may be affected by MMP-2 protein expression in HCT-116 cell. As shown in other data, Chuang C-H et al. claimed that quercetin metabolites can inhibit MMP-2 expression in A549 lung cancer cells, while S-C. Cheng et al. reported that quercetin could cause downregulation of ICAM-1 and MMP-9 in TNF-α-activated retinal pigment epithelial cells 33,38. Furthermore, Ghafouri-Fard et al. suggested that quercetin targeted inhibition in MMP-2, 7, 9 and 10 and iNOS in oral cancer 39. Taken together, our results demonstrate that quercetin in combination with NAC shows potent anti-migration and anti-invasion effects in both highly differentiated HT-29 and a highly aggressive HCT-116 cells, which are related to reducing the levels of iNOS, ICAM-1 and MMP-2 markers compared with quercetin treatment alone. Additionally, the GEPIA2 database further confirmed the gene expression of iNOS, ICAM-1, and MMP-2. This outcome demonstrates that COAD has an upregulation of these genes. In particular, elevated ICAM-1 and MMP-2 expression are associated with a poor prognosis in COAD patients. Taken together, these studies indicate that using a combination of quercetin and NAC may be a potential new treatment choice for colorectal cancer and merits further exploration.

## Conclusion

These results verify that, when used in combination with NAC, quercetin effectively suppresses migration and invasion through cellular oxidative stress (especially in iNOS) by suppressing ICAM-1 and MMP-2 proteins in colorectal cancer cells (CRC). Our study also suggests that the combination of quercetin and NAC treatment should be administered in the CRC progression stage.
